# Anomalous coloration in European pine marten *Martes martes* in Elba Island, Central Italy

**DOI:** 10.1002/ece3.8980

**Published:** 2022-06-06

**Authors:** Emiliano Manzo, Paola Bartolommei, Filippo Dell’Agnello, Roberto Cozzolino

**Affiliations:** ^1^ 582581 Fondazione Ethoikos Radicondoli Italy

**Keywords:** coat color, depigmentation, fur color, leucism, mustelid

## Abstract

Evidence of abnormal coloration in wild animals provides useful information to better understand its adaptive function and its impact on survival. For this reason, we need to know the frequency and distribution of these abnormal phenotypes in wild populations. Here, we report two records of hypopigmentation in European pine marten *Martes martes*, obtained during a camera‐trapping survey on Elba Island, Central Italy. We do not know what has caused anomalous coloration of pine marten on Elba Island, but it is possible that the inbreeding may have played a role in this isolated population. Although the light coloration certainly entails an increased visibility of pine martens, it is possible that the low predator pressure and the absence of other wild carnivore populations in our study could mitigate the mortality risk due to the light phenotype. The increased use of camera traps across the world can potentially facilitate the discovery of cases of anomalous colorations in wild populations, providing an unprecedented insight into the occurrence of this phenomenon in wild mammal species.

## Graphical abstract

Evidence of abnormal coloration in wild animals provides useful information to better understand their adaptive function and their impact on survival. For this reason, it is useful to know the frequency and distribution of these abnormal phenotypes in wild populations. Here, we report two records of hypopigmentation in European pine marten *Martes martes*, obtained during a camera‐trapping survey on Elba Island, Central Italy.

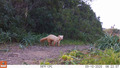

## 1 INTRODUCTION

Animal coloration can play an adaptive role in communication, concealment, sexual selection, and physiological function (Caro, 2005; Caro & Mallarino, 2020). In mammals, coat color is determined by the presence and the distribution of pigmentation (melanin) in the skin, hair, and eyes (Hofreiter & Schöneberg, 2010). Eumelanin and pheomelanin are two different forms of melanin responsible for black–brown coloration and red–yellow coloration, respectively (Gong et al., 2021). However, excessive or deficient melanin in part of or in the whole body can occasionally entail anomalous color. In accordance with Gong et al. (2021), anomalous coloration due to a deficiency of melanin can be classified as albinism, leucism, piebaldism, and dilution, although there is still some divergence between authors about the use of these definitions. As reported by Gong et al. (2021), albinism is characterized by a total lack of melanin, leading to de‐pigmented skin, fur, and eyes (Fertl & Rosel, 2002; Lucati & López‐Baucells, 2017). Leucism is caused by a partial or total absence of pigmentation throughout the whole body, but rarely affects hairless body parts, such as the nose and feet, and never affects the eyes, leading to de‐pigmented fur only (Abreu et al., 2013; Acevedo & Aguayo, 2008; Arriaga‐Flores et al., 2016; Lucati & López‐Baucells, 2017). Piebaldism is used when melanin is absent from some or all of the areas in which it is normally present, causing spotting patterns ranging from sparse white markings to total body discoloration, but rarely is accompanied by hypopigmented eyes (Abreu et al., 2013; Lamoreux et al., 2010; Lucati & López‐Baucells, 2017). Dilution is characterized by paler and more silvery coloration than normal due to insufficient pigmentation (Gong et al., 2021; Lamoreux et al., 2010). Abnormal colorations often occur in isolated and genetically homogenous populations as they are caused by single mutations in specific genes (Abreu et al., 2013; Hubbard et al., 2010). However, records of this phenomenon in wild populations are underestimated and often misclassified due to difficulties in capturing specimens and performing genetic testing (Gong et al., 2021).

In mammals, genetic anomalies in coat color are documented in most taxa (Abreu et al., 2013; Camargo et al., 2014; Cronemberger et al., 2018; Descalzo et al., 2021; Keener et al., 2011; McAlpine, 2021; Muller, 2017; Olson & Allen, 2019). The increase in camera trap use has recently been revealing new evidence on this phenomenon in mustelids too (Allen et al., 2019; Gong et al., 2021; Hofmeester et al., 2021; Olson & Allen, 2019; Scrich et al., 2019). Within Mustelids, leucism or albinism cases have been documented in wild populations of tayra *Eira barbara* (Mendes‐Pontes et al., 2020; Talamoni et al., 2017), neotropical otter *Lontra longicaudis* (Arriaga‐Flores et al., 2016), Eurasian otter *Lutra lutra* (Goncharuk et al., 2020), oriental small‐clawed otter *Aonyx cinereus* (Allen et al., 2019), fisher *Pekania pennanti* (Olson & Allen, 2019), Eurasian badger *Meles meles* (Hofmeester et al., 2021), and yellow‐throated marten *Martes flavigula* (Gong et al., 2021).

**FIGURE 1:**
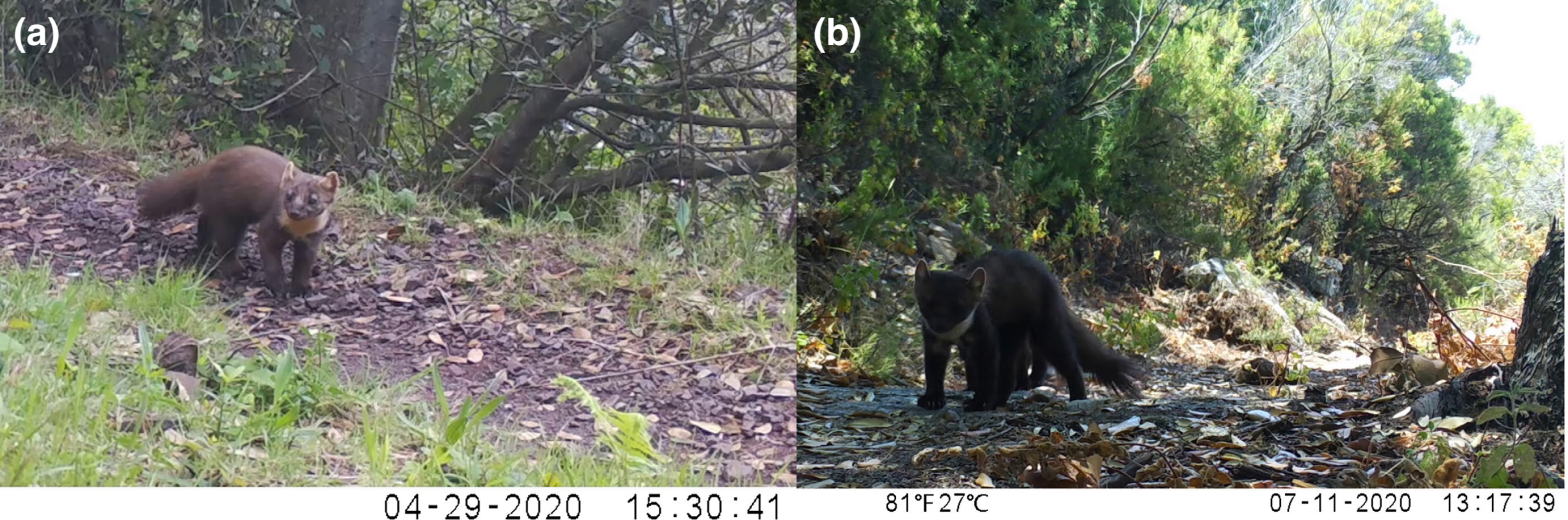
Camera trap images of regularly colored pine martens from the study area, showing the tawny coat with a creamy‐yellow throat patch: (a) longer and lighter winter fur; (b) shorter and darker summer fur

Here, we report evidence of hypopigmentation in the European pine marten *Martes martes*, an elusive medium‐sized mustelid distributed throughout much of Europe and northern and Central Asia, and inhabiting a variety of habitats (Aubry et al., 2012). In Italy, the species is present with a fragmented distribution in the forested areas of the peninsula, but it has been recently detected even in anthropic and cultivated areas (Balestrieri et al., 2010; Manzo et al., 2018). Insular populations occur in Sardinia, Sicily, and Elba (Genovesi & De Marinis, 2003). The coat color of pine marten ranges from to dark brown to tawny, with a creamy‐yellow throat patch and pale fur within ears (Genovesi & De Marinis, 2003; Figure 1). The fur changes seasonally, being longer and lighter in color in winter and becoming shorter and darker in summer (Figure 1). Geographical, seasonal, and age‐related variation in fur color, with a tendency toward partial lightening, have been reported for other representatives of the genus *Martes* Pinel 1792, such as sable *M*. *zibellina* (Safronov & Zakharov, 2014), Japanese marten *M*. *melampus* (Funakoshi et al., 2017), *M*. *flavigula* (Gong et al., 2021), and stone marten *M*. *foina* (Delibes & Amores, 1986; Masseti, 2009). However, anomalous coloration in European pine marten has never been documented in scientific literature.

## 2 MATERIALS AND METHODS

As a part of a larger study on European pine marten distribution on Elba island (42°47’12’’N, 10°16’28’’E), we conducted a camera‐trapping survey between February and July 2020. Elba Island is the largest island of the Tuscan Archipelago (Central Italy) with a total area of 223 km^2^. The island is characterized by high geomorphological heterogeneity and by an altitude ranging from sea level up to 1019 m a.s.l. (Monte Capanne), leading to the establishment of three distinct bioclimatic belts and a large vegetation diversity (Foggi et al., 2006). The climate is Mediterranean with mean yearly temperature of 16.5°C (min. 10°C in January, max. 24.5°C in July) and mean yearly precipitation of 595 mm (min. 13 mm in July, max. 86 mm in November). More than half of the island (127.4 km^2^) was designated as a National Park in 1996. The pine marten is the only wild carnivore species present on the island; however, the presence of domestic and feral cats on the island does not allow us to consider Elba Island a competitor‐free area. Currently available information about pine marten on Elba Island covers the origin, distribution, feeding habits, and spatiotemporal activity of the species (De Marinis & Masseti, 1993, 1995; Mori et al., 2021).

**FIGURE 2:**
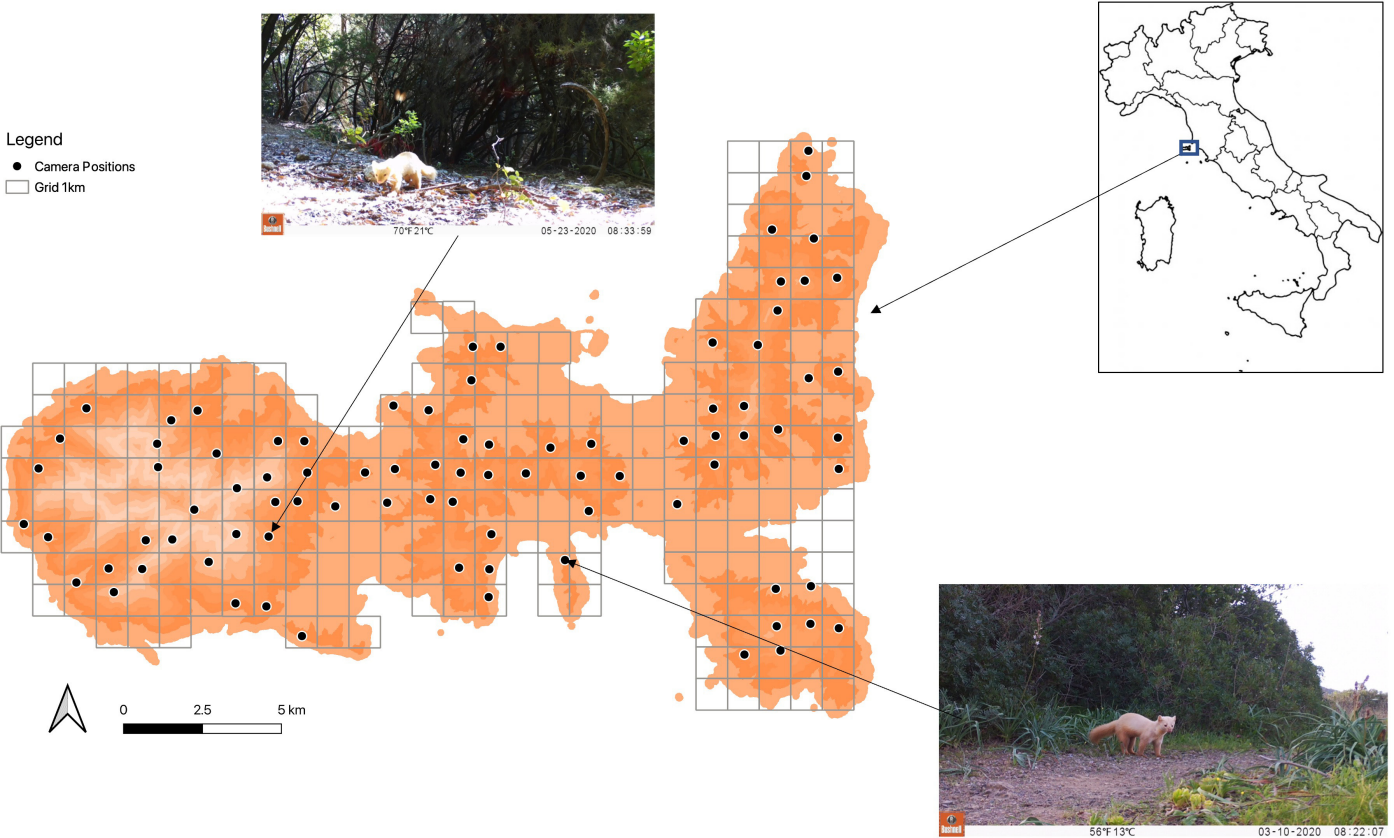
Map of Elba Island, Central Italy, showing the camera trap arrangement for the pine marten survey and the location of cameras recording the two videos of hypopigmented pine marten

We generated 1‐km^2^ grid cells over the entire island and then we fixed the centroids, which represent the ideal camera locations (Figure 2). We positioned 77 camera traps (Trophy cam HD Aggressor, No Glow, model 119877, Bushnell Outdoor Products), which remained in the field for 30 days. Camera traps were placed in the field as close as possible to the ideal locations, at sites where a suitable tree, shrub, or rock was available for mounting the camera and where the surrounding area was sufficiently open for the camera to have a clear view. Cameras were placed at 0–30 cm above the ground, unbaited, and set to record 30‐s‐long videos when triggered, with a 1‐min delay. We recorded a total of 365 marten videos in 55 of 77 sampling sites (71%) during a span of 2310 trap‐days.

### 2.1 Results

**FIGURE 3:**
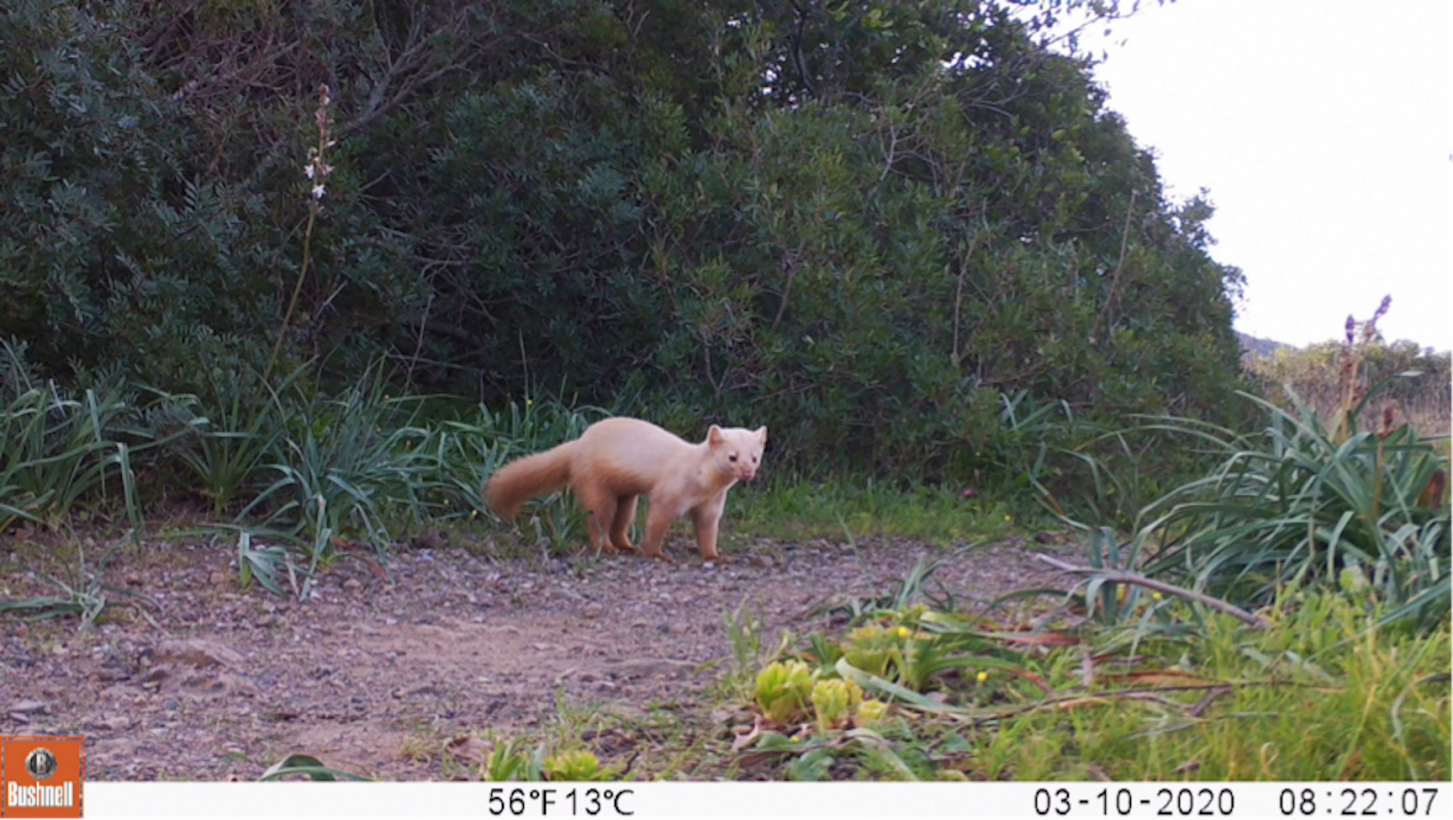
Anomalous colored pine marten recorded on March 10, 2020, at 08:22 h UTC + 1 at 42°45'14.0"N, 10°19'02.5"E Lacona location, Elba island, Italy

**FIGURE 4:**
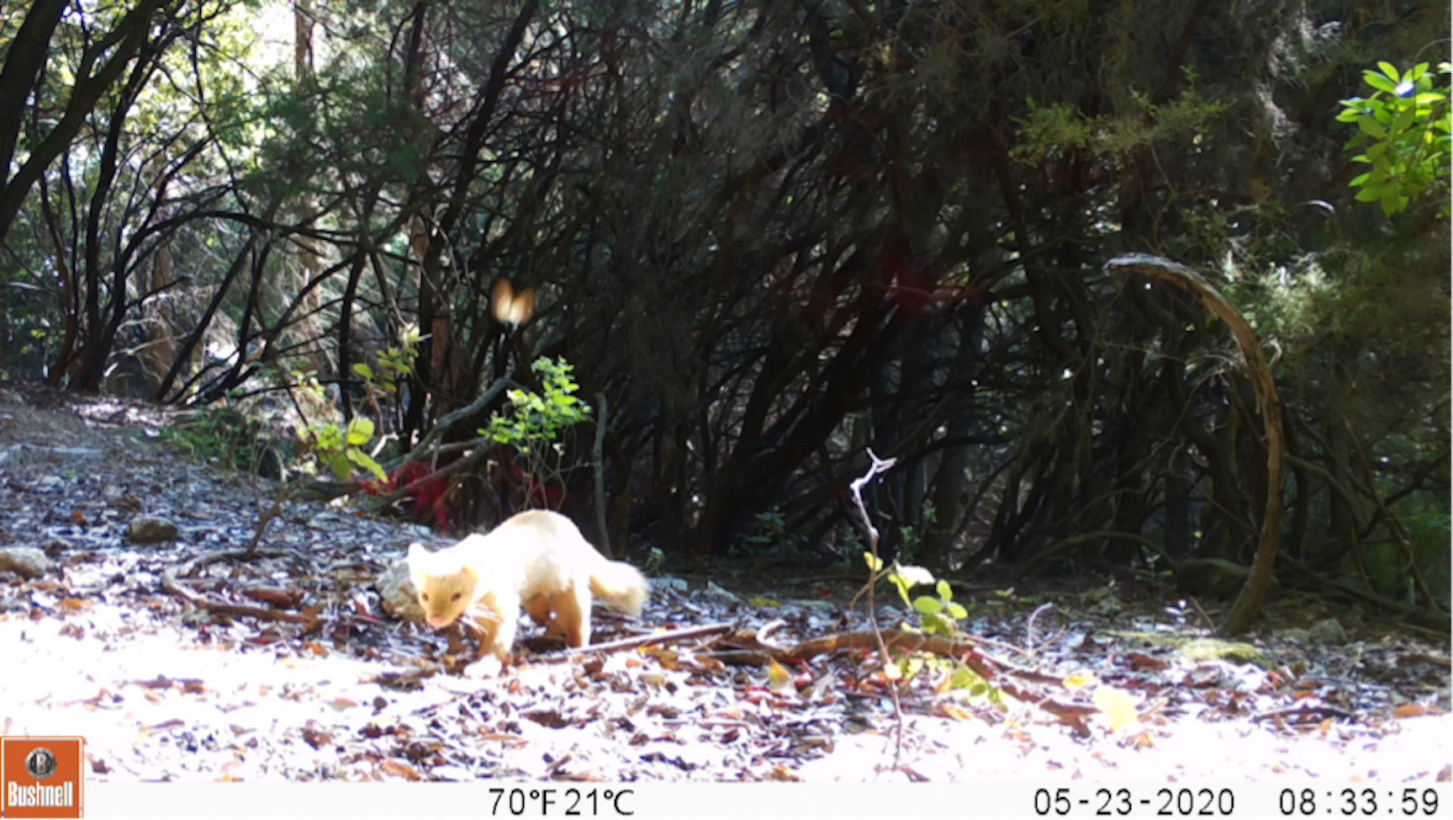
Anomalous colored pine marten recorded on May 23, 2020, at 08:34 h UTC + 1 at 42°45'39.7"N, 10°12'10.5"E Pieve di San Giovanni location, Elba island, Italy

On March 10, 2020, at 08:22 h UTC+1 and on May 23, 2020, at 08:34 h UTC+1, two videos of pine marten with anomalous coloration were recorded by camera traps located in two different parts of the island (42°45'14.0"N, 10°19'02.5"E; 42°45'39.7"N, 10°12'10.5"E, Figure 2). Frames from these videos can be seen in Figures 3 and 4. Subsequent investigation revealed that a pine marten with pale yellow coat had already been seen in the previous months by a local hunter, at the same site where we recorded the aforementioned May video. According to the morphological features (i.e., body shape) of the animals noticeable in the two videos, we could hypothesize that the records concern two different individuals. The distance between the two camera locations (about 9 km, Figure 2) might support this suggestion. Although there are no published studies on pine marten spatial distribution on Mediterranean islands, the distance between the two cameras was larger than the home range size and distance estimated for this species in Central Italy (Bartolommei, Gasperini, et al., 2016; Bartolommei, Manzo, et al., 2016). Both videos show adult pine martens (i.e., individuals older than 1 year) with a lack of pigmentation throughout the whole body including the nose and with black eye color (Figures 3 and 4). Although the hypopigmentation of the nose might suggest albinism (Fertl & Rosel, 2002; Gong et al., 2021), the eye color leads us to suppose that it could correspond to a case of leucism rather than albinism. In fact, the marten fur appears not to be totally depigmented and was pale yellow with an even lighter throat patch in the standard location (Figures 3 and 4).

## 3 DISCUSSION

Depigmentation has been often studied as a stage of the domestication process (Cieslak et al., 2011; Trut et al., 2009). However, the frequency of these color anomalies could be higher in small and isolated wild populations due to inbreeding or founder effect, which may increase the expression of recessive phenotypes (Abreu et al., 2013; Bensch et al., 2000; Hubbard et al., 2010; van der Geer, 2019). In Central Norway, Hofmeester et al. (2021) recorded the occurrence of leucistic badgers probably explainable as a result of the founder effect, since badgers colonized this topographically fragmented study area only recently. Anomalous coloration can be also associated with environmental factors, such as pollution, environmental alterations, low‐quality habitat, and diet (Mendes‐Pontes et al., 2020; Olson & Allen, 2019; Peles et al., 1995). Although we do not know what has caused anomalous coloration of pine marten on Elba Island, we hypothesize that the inbreeding may have played a role in this isolated population.

Leucism and albinism might have negative consequences for the affected individuals in wild populations, such as reduced mating opportunities, altered communication, increased detection by both predators and preys, thermoregulatory consequences, and pathologies related to the genetic anomaly (Abreu et al., 2013; Caro & Mallarino, 2020; Olson & Allen, 2019). These disadvantages decrease the chance of a point mutation spreading through the population, and thus could explain the rarity of the anomalous colored individuals in small and isolated wild populations. Nonetheless, Hofmeester et al. (2021) state that the presence of leucistic adult badgers casts doubt on the actual impact of leucism on hunting success and survival. Arriaga‐Flores et al. (2016) also point out that the leucistic neotropical otters they recorded were full‐grown adults with no sign of impeded development. In effect, some authors suggest that depigmented and normally colored individuals do not differ in terms of survival in cryptic or nocturnal species and in those that have few predators (Abreu et al., 2013; Hubbard et al., 2010; Peles et al.,^1995^). Allen et al. (2019) even hypothesize that, occasionally, hypopigmented individuals can have evolutionary advantages wherever they are prevalent in the population. Actually, leucism seems to be common in tayras based on reports from Guyana, Bolivia, and Brazil (Mendes‐Pontes et al., 2020; Scrich et al., 2019; Talamoni et al., 2017). Mendes‐Pontes et al. (2020) documented leucistic tayras in a population of Guyana considered neither small nor isolated, where hypopigmented individuals did not appear to face the disadvantages described in the literature. We cannot define if the light coloration causes an adaptive disadvantage for pine martens on Elba Island, even if both videos show individuals that survived to maturity. Although the light coloration certainly entails an increased visibility of pine martens, it is possible that the low predator pressure and the absence of other wild carnivore populations in our study area could mitigate the mortality risk due to the anomalous coloration.

As pointed out by several authors (Hofmeester et al., 2021; Olson & Allen, 2019), the increasing number of wildlife camera‐trapping projects across the world can potentially provide a greater number of records of anomalous coat coloration in mammals, expanding knowledge on the occurrence and distribution of this phenomenon. A systematic recording of abnormal coloration in wild species may help researchers get a deeper insight into the adaptive significance of anomalous phenotypes and their impact on survival. We encourage researchers to report records of abnormal color conditions in wildlife.

## AUTHOR CONTRIBUTIONS

**Emiliano Manzo:** Conceptualization (lead); Investigation (equal); Methodology (equal); Project administration (equal); Writing – original draft (lead); Writing – review & editing (lead). **Paola Bartolommei:** Conceptualization (lead); Investigation (equal); Methodology (equal); Writing – review & editing (equal); Writing – original draft (equal). **Filippo Dell'Agnello:** Conceptualization (equal); Investigation (equal); Methodology (equal); Writing – review & editing (equal). **Roberto Cozzolino:** Conceptualization (equal); Funding acquisition (lead); Project administration (lead); Writing – review & editing (equal).

## CONFLICT OF INTEREST

The authors declare no competing interests.

## Data Availability

Data sharing is not applicable to this article as no datasets were generated or analyzed during the current study.

## References

[ece38980-bib-0001] Abreu, M. S. L. , Machado, R. , Barbieri, F. , Freitas, N. S. , & Oliveira, L. R. (2013). Anomalous colour in Neotropical mammals: A review with new records for *Didelphis* sp. (Didelphidae, Didelphimorphia) and *Arctocephalus australis* (Otariidae, Carnivora). Brazilian Journal of Biology, 73, 185–194. 10.1590/S1519-69842013000100020 23644801

[ece38980-bib-0002] Acevedo, J. , & Aguayo, M. (2008). Leucistic South American sea lion in Chile, with a review of anomalously color in otariids. Revista De Biología Marina Y Oceanografía, 43(2), 413–417. 10.4067/S0718-19572008000200017

[ece38980-bib-0003] Allen, M. L. , Sibarani, M. C. , & Utoyo, L. (2019). The first record of a wild hypopigmented Oriental small‐clawed otter (*Aonyx cinereus*). International Journal, 21, 201904. 10.30427/ecotrop201905

[ece38980-bib-0004] Arriaga‐Flores, J. C. , Rodríguez‐Ruíz, E. R. , Gallo‐Reynoso, J. P. , & Castro‐Arellano, I. (2016). Leucism in neotropical otters (*Lontra longicaudis annectens*) from Mexico. The Southwestern Naturalist, 61, 63–68. 10.1894/0038-4909-61.1.63

[ece38980-bib-0005] Aubry, K. B. , Zielinski, W. J. , Raphael, M. G. , Proulx, G. , & Buskirk, S. W. (2012). Biology and conservation of martens, sables and fishes: a new synthesis (pp. 608). Cornell University Press.

[ece38980-bib-0006] Balestrieri, A. , Remonti, L. , Ruiz‐González, A. , Gomez‐Moliner, B. J. , Vergara, M. , & Prigioni, C. (2010). Range expansion of the pine marten (*Martes martes*) in an agricultural landscape matrix (NW Italy). Mammalian Biology, 75, 412–419. 10.1016/j.mambio.2009.05.003

[ece38980-bib-0007] Bartolommei, P. , Gasperini, S. , Manzo, E. , Natali, C. , Ciofi, C. , & Cozzolino, R. (2016). Genetic relatedness affects socio‐spatial organization in a solitary carnivore, the European pine marten. Hystrix, the Italian Journal of Mammalogy, 27(2), 222–224. 10.4404/hystrix-27.2-11876

[ece38980-bib-0008] Bartolommei, P. , Manzo, E. , & Cozzolino, R. (2016). Seasonal spatial behaviour of pine marten *Martes martes* in a deciduous oak forest of central Italy. Mammal Research, 61(4), 319–326. 10.1007/s13364-016-0278-9

[ece38980-bib-0009] Bensch, S. , Hansson, B. , Hasselquist, D. , & Nielsen, B. (2000). Partial albinism in a semi‐isolated population of Great Reed Warblers. Hereditas, 133, 167–170. 10.1111/j.1601-5223.2000.t01-1-00167.x 11338429

[ece38980-bib-0010] Camargo, E. R. , Cornejo‐Latorre, C. , & Álvarez‐Castañeda, S. T. (2014). First Record of Leucism in the Genus *Peromyscus* (Mammalia: Rodentia). Western North American Naturalist, 74(3), 366–368. 10.3398/064.074.0301

[ece38980-bib-0011] Caro, T. (2005). The adaptive significance of coloration in mammals. BioScience, 55, 125–136.

[ece38980-bib-0012] Caro, T. , & Mallarino, R. (2020). Coloration in mammals. Trends in Ecology & Evolution, 35, 357–366. 10.1016/j.tree.2019.12.008 31980234 PMC10754262

[ece38980-bib-0013] Cieslak, M. , Reissmann, M. , Hofreiter, M. , & Ludwig, A. (2011). Colours of domestication. Biogical Reviews, 86, 885–899. 10.1111/j.1469-185X.2011.00177.x 21443614

[ece38980-bib-0014] Cronemberger, C. , Pereira, F. A. , Bacellar, A. E. F. , & Silva, L. G. (2018). First record of leucism in puma from Serra dos Órgãos National Park, Brazil. Cat News, 68, 38–40.

[ece38980-bib-0015] De Marinis, A. M. , & Masseti, M. (1993). Distribution of the pine marten *Martes martes* L., 1758 (Mammalia, Carnivora), on the Island of Elba, northern Tirrenian sea. Supplemento Alle Ricerche Di Biologia Della Selvaggina, 21, 263–267.

[ece38980-bib-0016] De Marinis, A. M. , & Masseti, M. (1995). First data on winter feeding of pine marten *Martes martes* in the island of Elba (Northern Tyrrhenian sea, Italy). Vie Milieu, 46(3–4), 382–383.

[ece38980-bib-0017] Delibes, M. , & Amores, F. (1986). The Stone Marten *Martes foina* (Erxleben, 1777) (Mammalia, Carnivora) from Ibiza (Pitiusic, Balearic Islands). Miscellania Zoologica, 10, 335–345.

[ece38980-bib-0018] Descalzo, E. , Nájera, F. , Mata‐Huete, M. , Sánchez, J. F. , Cáceres‐Urones, J. , Jiménez, J. , Delibes‐Mateos, M. , Díaz‐Ruiz, F. , & Ferreras, P. (2021). First records of anomalous colouration in the Egyptian mongoose (*Herpestes ichneumon*). Galemys, 33, 57–60. 10.7325/Galemys.2021.N6

[ece38980-bib-0019] Fertl, D. , & Rosel, P. (2002). Albinism. In W. F. Perrin , B. Würsig , & J. G. M. Thewissen (Eds.), Encyclopedia of marine mammals (pp. 16–18). Academic Press.

[ece38980-bib-0020] Foggi, B. , Cartei, L. , Pignotti, L. , Signorini, M. A. , Viciani, D. , Dell’Olmo, L. , & Menicagli, E. (2006). Il paesaggio vegetale dell’Isola d’Elba (Arcipelago Toscano). Studio Fitosociologico E Cartografico. Fitosociologia, 43(1), 3–95.

[ece38980-bib-0021] Funakoshi, K. , Nagasato, A. , Takenouchi, S. , Kannonji, R. , Kikusui, M. , Uchihara, A. , & Tamai, K. (2017). Annual molting cycle and photoperiods that affect seasonal coat color changes in the Japanese marten (*Martes melampus*). Mammal Study, 42, 209–218.

[ece38980-bib-0022] Genovesi, P. , & De Marinis, A. M. (2003). *Martes martes* – Distribuzione geografica. In L. Boitani , S. Lovari , & T. A. Vigna (Eds.), Fauna d’Italia, Vol. XXXVIII, Mammalia III, Carnivora‐Artiodactyla (pp. 104–113). Calderini.

[ece38980-bib-0023] Goncharuk, M. S. , Voloshina, I. V. , Aramilev, V. V. , Shurygina, A. A. , & Kerley, L. L. (2020). Recent observations of Eurasian otter *Lutra lutra*, including white‐coated individuals, in the southern Sikhote Alin, the Russian far east. IUCN Otter Specialist Group Bulletin, 37, 147–157.

[ece38980-bib-0024] Gong, Y. , Zhao, G. , Yang, H. , Li, Y. , Tan, M. , Wang, N. , Ge, J. , Yang, H. , & Feng, L. (2021). Prevalence of Varied Coat Coloration in a Yellow‐Throated Marten (*Martes flavigula*) Population. Animals, 11, 2838. 10.3390/ani11102838 34679859 PMC8532798

[ece38980-bib-0025] Hofmeester, T. R. , Thorsen, N. H. , Linnell, J. D. C. , & Odden, J. (2021). Camera trap records of leucistic Eurasian badgers (*Meles meles*) in central Norway. Ecology and Evolution, 11, 12902–12907. 34646442 10.1002/ece3.8052 PMC8495824

[ece38980-bib-0026] Hofreiter, M. , & Schöneberg, T. (2010). The genetic and evolutionary basis of colour variation in vertebrates. Cellular and Molecular Life Sciences, 67, 2591–2603. 10.1007/s00018-010-0333-7 20229234 PMC11115542

[ece38980-bib-0027] Hubbard, J. K. , Uy, J. A. C. , Hauber, M. E. , Hoekstra, H. E. , & Safran, R. J. (2010). Vertebrate pigmentation: From underlying genes to adaptive function. Trends in Genetics, 26, 231–239. 10.1016/j.tig.2010.02.002 20381892

[ece38980-bib-0028] Keener, W. , Szczepaniak, I. , Adam, Ü. , & Webber, M. (2011). First records of anomalously white harbour porpoises (*Phocoena phocoena*) from the Pacific Ocean. Journal of Marine Animals and their Ecology, 4, 19–24.

[ece38980-bib-0029] Lamoreux, M. L. , Delmas, V. , Larue, L. , & Bennett, D. C. (2010). The colors of mice: A model genetic network (pp. 312). Wiley.

[ece38980-bib-0030] Lucati, F. , & López‐Baucells, A. (2017). Chromatic disorders in bats: A review of pigmentation anomalies and the misuse of terms to describe them. Mammal Review, 47(2), 112–123. 10.1111/mam.12083

[ece38980-bib-0031] Manzo, E. , Bartolommei, P. , Giuliani, A. , Gentile, G. , Dessì‐Fulgheri, F. , & Cozzolino, R. (2018). Habitat selection of European pine marten in Central Italy: From a tree dependent to a generalist species. Mammal Research, 63, 357–367. 10.1007/s13364-018-0374-0

[ece38980-bib-0032] Masseti, M. (2009). Mammals of the Mediterranean islands: homogenisation and the loss of biodiversity. Mammalia, 73(3), 169–202. 10.1515/MAMM.2009.029

[ece38980-bib-0033] McAlpine, D. F. (2021). Further occurrences of melanism in a northern, peripheral, population of Bobcat (*Lynx rufus*). Canadian Field‐Naturalist, 135(1), 52–57. 10.22621/cfn.v135i1.2449

[ece38980-bib-0034] Mendes‐Pontes, A. R. , Da Silva Júnior, A. P. , & Chivers, D. (2020). The occurrence of leucism in groups of tayras *Eira barbara* Linnaeus 1758 on the Guyana shield. Ecoscience, 27, 295–301. 10.1080/11956860.2020.1804724

[ece38980-bib-0035] Mori, E. , Fedele, E. , Greco, I. , Giampaoli Rustichelli, M. , Massolo, A. , Miniati, S. , Puppo, F. , Santini, G. , & Zaccaroni, M. (2021). Spatiotemporal activity of the pine marten *Martes martes*: Insights from an island population. Ecological Research, 37, 102–114.

[ece38980-bib-0036] Muller, Z. (2017). White giraffes: The first record of vitiligo in a wild adult giraffe. African Journal of Ecology, 55, 118–123. 10.1111/aje.12323

[ece38980-bib-0037] Olson, L. O. , & Allen, M. L. (2019). A leucisitic fisher (*Pekania pennanti*) and the prevalence of Leucism in wild carnivores. American Midland Naturalist, 181, 133–138. 10.1674/0003-0031-181.1.133

[ece38980-bib-0038] Peles, J. D. , Lucas, M. F. , & Barrett, G. W. (1995). Population dynamics of agouti and albino meadow voles in high‐quality, grassland habitats. Journal of Mammalogy, 76, 1013–1019. 10.2307/1382595

[ece38980-bib-0039] Safronov, V. M. , & Zakharov, E. S. (2014). Changes in fur color in different age groups of the sable (*Martes zibellina*, Carnivora, Mustelidae) in the middle Aldan River basin. Biology Bulletin, 41(9), 817–821. 10.1134/S1062359014090076

[ece38980-bib-0040] Scrich, V. M. , Pônzio, M. C. , Pasqualotto, N. , Rodrigues, T. F. , Paolino, R. M. , & Chiarello, A. G. (2019). Occurrence of tayras (*Eira barbara* Linnaeus, 1758) with anomalous coloration in Cerrado remnants in the state of São Paulo, Brazil. Biota Neotropica, 67(4), e20180680. 10.1590/1676-0611-bn-2018-0680

[ece38980-bib-0041] Talamoni, S. , Viana, P. I. M. , Costa, C. G. , Palú, L. , Oliveira, R. B. , & Pessôa, L. M. (2017). Occurrence of leucism in *Eira barbara* (Carnivora, Mustelidae) in Brazil. Biota Neotropica, 17(3), e20170328. 10.1590/1676-0611-bn-2017-0328

[ece38980-bib-0042] Trut, L. N. , Oskina, I. , & Kharlamova, A. (2009). Animal evolution during domestication: The domesticated fox as a model. BioEssays, 31, 349–360. 10.1002/bies.200800070 19260016 PMC2763232

[ece38980-bib-0043] van der Geer, A. A. E. (2019). Effect of isolation on coat colour polymorphism of Polynesian rats in Island Southeast Asia and the Pacific. PeerJ, 7, e6894. 10.7717/peerj.6894 31119086 PMC6511229

